# Cardiovascular adverse events associated with targeted therapies for multiple myeloma: a pharmacovigilance study

**DOI:** 10.3389/fimmu.2024.1400101

**Published:** 2024-09-26

**Authors:** Yanli Zhang, Chang Shan, Xinxin Zhang, Ying Liu, Yunlong Xia, Yanfeng Wang

**Affiliations:** ^1^ Department of Cardiology, The First Affiliated Hospital of Dalian Medical University, Dalian, Liaoning, China; ^2^ Department of Comprehensive Oncology, National Cancer Center/National Clinical Research Center for Cancer/Cancer Hospital, Chinese Academy of Medical Sciences and Peking Union Medical College, Beijing, China

**Keywords:** multiple myeloma, cardio-oncology, cardiovascular adverse event, targeted therapy, cardiovascular safety

## Abstract

**Introduction:**

Multiple myeloma (MM) is a leading cause of hematopoietic cancer-related mortality, accounting for 20% of deaths. MM-targeted therapies have demonstrated efficacy, and since 2015, the United States Food and Drug Administration (FDA) has approved five targeted drugs. However, their cardiovascular safety has not been comprehensively evaluated.

**Objective:**

This study aimed to investigate the association between MM-targeted therapy and cardiovascular adverse events (AEs).

**Methods:**

Disproportionality analysis was conducted on reports from the FDA AE Reporting System database from 2014 to the second quarter of 2023. Cardiovascular AEs were grouped into nine narrow categories using the Standardized Medical Dictionary for Regulatory Activities Queries (SMQs).

**Results:**

A total of 3,228 cardiovascular AE cases involving MM-targeted therapy were extracted and analyzed. Significant disproportionality was identified for daratumumab, elotuzumab, and isatuximab. Among the nine narrow SMQ categories, the three most reported cardiovascular AEs were cardiomyopathy, cardiac arrhythmias, and embolic and thrombotic events. Noninfectious myocarditis/pericarditis, cardiac arrhythmias, and embolic and thrombotic events exhibited the strongest signal strengths. The cardiovascular AE risk was higher within the first month and gradually decreased thereafter; however, it increased rapidly again after 1 year. This trend was observed for all cardiovascular AEs. The Kaplan–Meier curve and the log-rank test revealed that isatuximab and elotuzumab exhibited a significantly lower probability of cardiovascular AEs than daratumumab (*p* < 0.001).

**Conclusions:**

MM-targeted therapy is significantly associated with an increased risk of previously unknown cardiovascular AE profiles, with the range and onset differing among various drugs, thereby warranting specific monitoring and appropriate management.

## Introduction

Multiple myeloma (MM), a clonal plasma cell dysplasia, ranks as the second most prevalent hematological malignancy, accounting for 20% of hematopoietic cancer-related deaths (1). Despite being incurable, MM diagnosis and treatment have witnessed remarkable improvements, with the aid of novel agents and sophisticated laboratory methods. Since 2015, the United States Food and Drug Administration (FDA) has approved several drugs for the treatment of MM, including proteasome inhibitors, selective inhibitors of nuclear export, monoclonal antibodies, and antibody–drug conjugates. Among these, monoclonal antibodies such as daratumumab (2), isatuximab (targeting CD38) (3), elotuzumab (which activates natural killer cells) (4), belantamab mafodotin [targeting B-cell maturation antigen (BCMA)] (5), and teclistamab (targeting CD3/BCMA) (6) have demonstrated considerable efficacy in prolonging survival and enhancing the quality of life of patients with MM. For example, the CASTOR trial, one of the largest trials for daratumumab, reported that the median overall survival with daratumumab plus bortezomib and dexamethasone (D-Vd) was 49.6 months, compared to 38.5 months for patients receiving bortezomib and dexamethasone alone. This demonstrated that D-Vd significantly prolonged the survival of patients with relapsed or refractory MM (7). The POLLUX study also demonstrated positive results with daratumumab plus lenalidomide and dexamethasone (D-Rd) compared with lenalidomide plus dexamethasone alone (Rd). The median survival duration for patients who received D-Rd was 67.6 months, compared to 51.8 months for those receiving Rd (8). Based on these clinical trial data and supporting evidence, the National Comprehensive Cancer Network Panel has classified daratumumab as a category 1, preferred option for the treatment of patients with relapsed/refractory MM (9).

However, it is important to recognize that many MM drugs have been linked to cardiovascular adverse events (AEs). Proteasome inhibitors, such as bortezomib and carfilzomib, and immunomodulatory drugs, including lenalidomide, pomalidomide, and thalidomide, may induce thrombosis, hypertension, heart failure, and atrial fibrillation (10–12). Moreover, limited published evidence is available regarding the cardiovascular AE profiles of the five newly approved target agents. Given the recent increase in the use of these drugs, our study aimed to analyze the reported cardiovascular AEs associated with approved MM-targeted therapies.

## Methods

### Data source and extraction

The United States FDA Adverse Event Reporting System (FAERS) database is a cost-free postmarketing safety surveillance system that collects millions of spontaneous AE reports from healthcare professionals, patients, and drug manufacturers around the world. FAERS functions as a vital repository, providing insights into real-world experiences and potential risks associated with medications and medical products post-market release. The extensive volume of data gathered at a national level from a diverse and extensive population, often under conditions that may not be represented in controlled clinical trials, renders FAERS exceptionally useful and effective for conducting pharmacovigilance studies.

In the FAERS, AEs are coded using preferred terms (PTs) based on the Medical Dictionary for Regulatory Activities (MedDRA) (version 25.1). These PTs may be related to various high-level terms, high-level group terms, and system-organ classes. To enhance precision, PTs encompassing symptoms, signs, investigations, and relevant diagnoses were systematically categorized using Standardized MedDRA Queries (SMQs) to define specific medical conditions of interest. In this study, cardiovascular AEs were classified into nine distinct SMQs (cardiac arrhythmia, cardiac failure, cardiomyopathy, embolic and thrombotic events, hypertension, ischemic heart disease, noninfectious myocarditis/pericarditis, pulmonary hypertension, and torsade de pointes/QT prolongation) (13).

Five commonly used target agents that gained marketing authorization from the FDA were selected to treat MM. The labels used by the National Center for Biotechnology Information were used to describe them, these being “daratumumab,” “elotuzumab,” “isatuximab,” “belantamab,” and “teclistamab.” Information on patient demographics and administrative information, drug/biological information, AEs, patient outcomes, reporting source, reporting drug therapy start and end dates, and indications was extracted.

The time span of the data was set between 2014 and the second quarter (Q2) of 2023. Only AEs related to drugs labeled as “Primary Suspect” were collected. To ensure the accuracy of the results, the principle of general exclusion was applied, which involved removal of duplications of the same CASEID, and filtration of inaccurate statistics. The onset was defined as the interval between the initiation date of targeted therapy and the date of onset of the cardiovascular AE. Reports lacking information on the “drug start date,” “case event date,” or cases in which the drug’s start date postdated the onset date of AEs were considered anomalous and excluded from analysis.

### Statistical analysis

Disproportionality analysis, commonly referred to as case–noncase analysis, is a method that is frequently used in pharmacovigilance studies, employing a two-by-two contingency table (**Table 1**) (13, 14). Two specific indices, the reporting odds ratio (ROR) and information components (ICs), were calculated to identify potential associations between the drugs and cardiovascular AEs. Statistical shrinkage transformation was applied to ensure robust results. The respective calculation formulas for the ROR and IC are as follows (15):


$$\text{ROR}=\frac{{\text{N }}_{\text{observed}}+0.5}{{\text{N}}_{\text{expected}}+0.5}$$

$$\text{IC}=\log_{2}(\frac{{\text{N}}_{\text{observed}}+0.5}{{\text{N}}_{\text{expected}}+0.5})$$

$${\text{N}}_{\text{expected}}={\text{N}}_{\text{drug}}*{\text{N}}_{\text{event}}/{\text{N}}_{\text{total}}$$

**Table 1:** Disproportionality analysis based on a two-by-two contingency table.

	Target adverse events	Other adverse events	Total
Targeted drug	a (N_observed_)	b	N_drug_ = a+b
Other drugs	c	d	c+d
Total	N_event_ =a+c	b+d	N_total_ =a+b+c+d

where N_observed_ is the observed number of reports of target drug-related AEs, N_expected_ is the expected number of reports of target drug-related AEs, N_drug (a+b)_ is the total number of reports of the target drug, N_event (a+c)_ is the total number of target AEs reports, and N_total (a+b+c+d)_ is the total number of reports in the database. The calculation formulas for the 95% confidence level interval (CI) of the ROR and IC are as follows:


$$\text{ROR }95\text{\%CI}={\text{e}}^{\ln\left(\text{ROR}\right)\;\pm \;1.96\sqrt{\frac{1}{\text{a}}\;+\;\frac{1}{\text{b}}\;+\;\frac{1}{\text{c}}\;+\;\frac{1}{\text{d}}}}$$

$${\text{IC}}_{025}=\text{IC}-3.3\times {\left({\text{N}}_{\text{observed}}+0.5\right)}^{-0.5}-2\times {\left({\text{N}}_{\text{observed}}+0.5\right)}^{-1.5}$$

$${\text{IC}}_{975}=\text{IC}+2.4\times {\left({\text{N}}_{\text{observed}}+0.5\right)}^{-0.5}-0.5\times {\left({\text{N}}_{\text{observed}}+0.5\right)}^{-1.5}$$

A potential signal was considered statistically significant if the lower limit of the 95% CI for ROR was greater than one (ROR_025_ > 1), or the lower limit of the 95% CI for IC exceeded zero (IC_025_ > 0). All analyses were performed using SAS version 9.4 (SAS Institute Inc., Cary, NC, United States).

To assess the trend of cardiovascular AE associated with targeted therapy drugs, Kaplan–Meier curves were applied to represent the cumulative cardiovascular AEs over time. We defined the reported cardiovascular AEs as the primary outcome, and pre-specified endpoints were recorded with the onset. The Kruskal–Wallis test was utilized to determine whether a statistically significant difference existed regarding the onset of specific target therapies. Statistical significance was set at *p* < 0.05.

## Results

### Descriptive analysis

**Figure 1:**
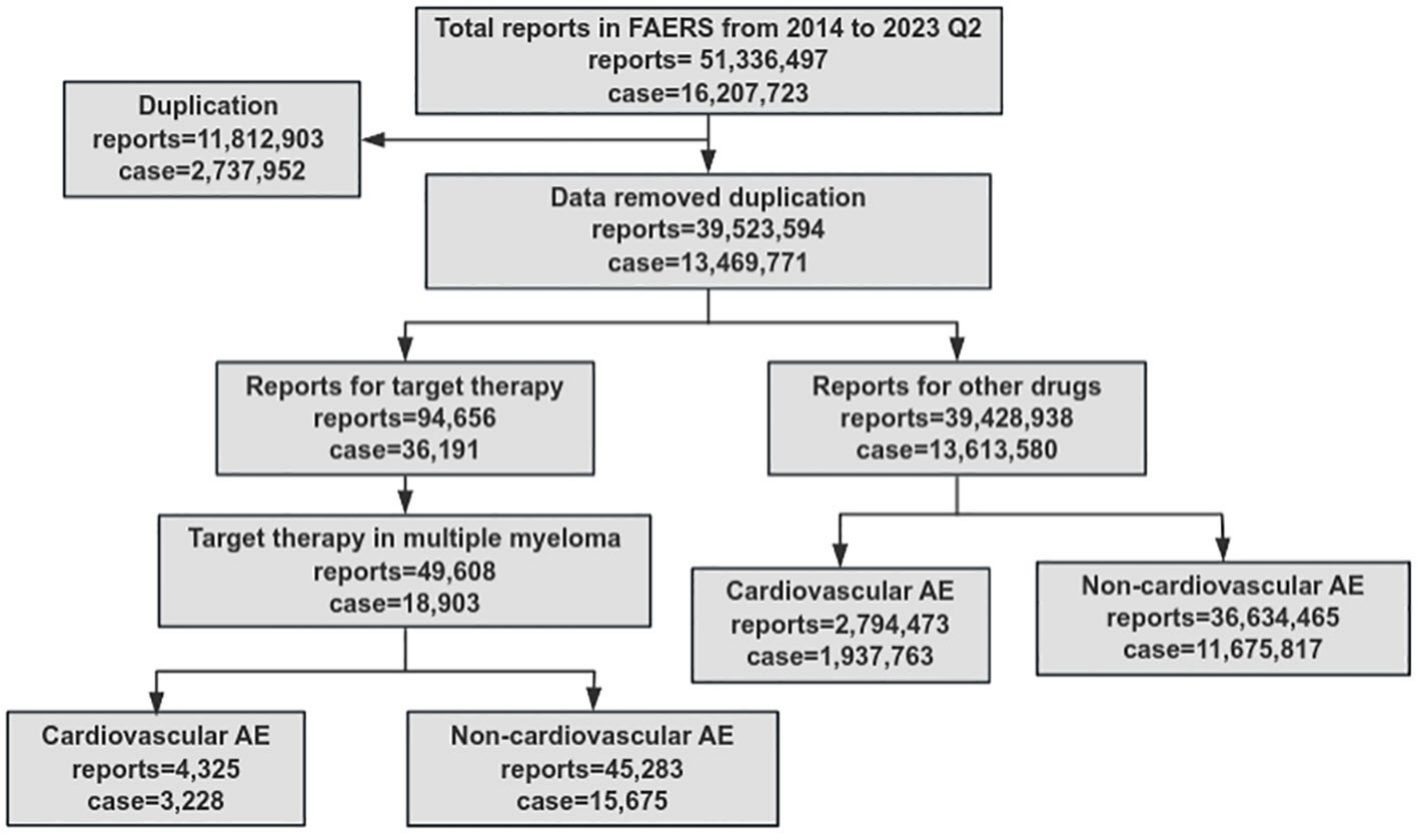
Flow diagram illustrating data filtering and categorizing.

A total of 51,336,497 AEs were reported, and 16,207,723 cases were identified from the FAERS database, between 2014 and 2023 Q2. Health professionals comprised the predominant source (68.8%) of the reports. After filtering and categorizing the data, the number of cases related to cardiovascular AEs associated with MM-targeted therapy was 3,228, while the number of non-cardiovascular AEs was 15,675 (**Figure 1**). The occurrence of AEs and cardiovascular disease increased annually owing to the ubiquitous use of targeted therapy.

Additionally, we examined the characteristics of patients with cardiovascular and overall AEs (**Table 2**). Regardless of whether patients had a non-classical definition of sex or some sex data were lost, men (51.9%) were more susceptible to AEs with MM-targeted therapy than women. The proportion of cardiovascular AEs in the ≥65-year-old subgroup was significantly higher than that of the <65-year-old subgroup (52.5% vs. 26.6%).

**Table 2:** Clinical characteristics of cardiovascular AEs reports for MM-targeted therapy.

Characteristics	Cardiovascular AEs	Overall AEs
Sex, *n* (%)	3,228	18,903
Female	1,174 (36.4)	6,774 (35.8)
Male	1,675 (51.9)	9,054 (47.9)
Unknown	379 (11.7)	3,075 (16.3)
Age (years), *n* (%)		
<18	94 (2.9)	767 (4.1)
18 to <65	766 (23.7)	5,115 (27.1)
65 to <75	982 (30.4)	4,460 (23.6)
≥75	713 (22.1)	3,160 (16.7)
Unknown	673 (20.9)	4,867 (28.5)
Outcome, *n* (%)		
Non-serious outcome	153 (4.7)	1,309 (6.9)
Serious outcome	3,075 (95.3)	17,594 (93.1)
Death	582 (18.0)	3,206 (17.0)
Lift-threatening	266 (8.2)	1,073 (5.7)
Hospitalization	1,364 (42.3)	6,704 (35.5)
Disability	7 (0.2)	86 (0.4)
Congenital anomalies	2 (0.1)	3 (0.1)
Other serious outcomes	854 (26.5)	6,510 (34.4)
Reporters, *n* (%)		
Health professional	2,222 (68.8)	11,773 (62.3)
Non-health professional	236 (7.3)	1,310 (6.9)
Others	770 (23.9)	5,820 (30.8)
Reporting countries, *n* (%)		
America (USA)	908 (28.1)	5,209 (27.6)
France	372 (11.5)	2,179 (11.5)
Japan	315 (9.8)	1,795 (9.5)
Germany	296 (9.2)	2,066 (10.9)
United Kingdom	134 (4.2)	716 (3.8)
Spain	134 (4.2)	987 (5.2)
Reporting year, *n* (%)		
2023 (Q1–Q2)	606 (18.8)	4,638 (24.5)
2022	642 (19.9)	4,348 (23.0)
2021	458 (14.2)	2,737 (14.5)
2020	443 (13.7)	2,120 (11.2)
2019	353 (10.9)	1,723 (9.1)
2018	310 (9.6)	1,412 (7.5)
2017	216 (6.7)	1,009 (5.3)
2016	151 (4.7)	610 (3.2)
2015	27 (0.8)	147 (0.8)
2014	22 (0.7)	159 (0.8)

a AEs, adverse events; MM, multiple myeloma.

Cardiovascular AEs were commonly associated with serious outcomes in most patients with AEs (95.3%). Among these serious outcomes, hospitalization (42.3%) and death (18.0%) were frequently reported. Considering nationality, the majority of cases were American (28.1%), French (11.5%), Japanese (9.8%), German (9.2%), British (4.2%), and Spanish (4.2%).

### Disproportionality analysis

**Figure 2:**
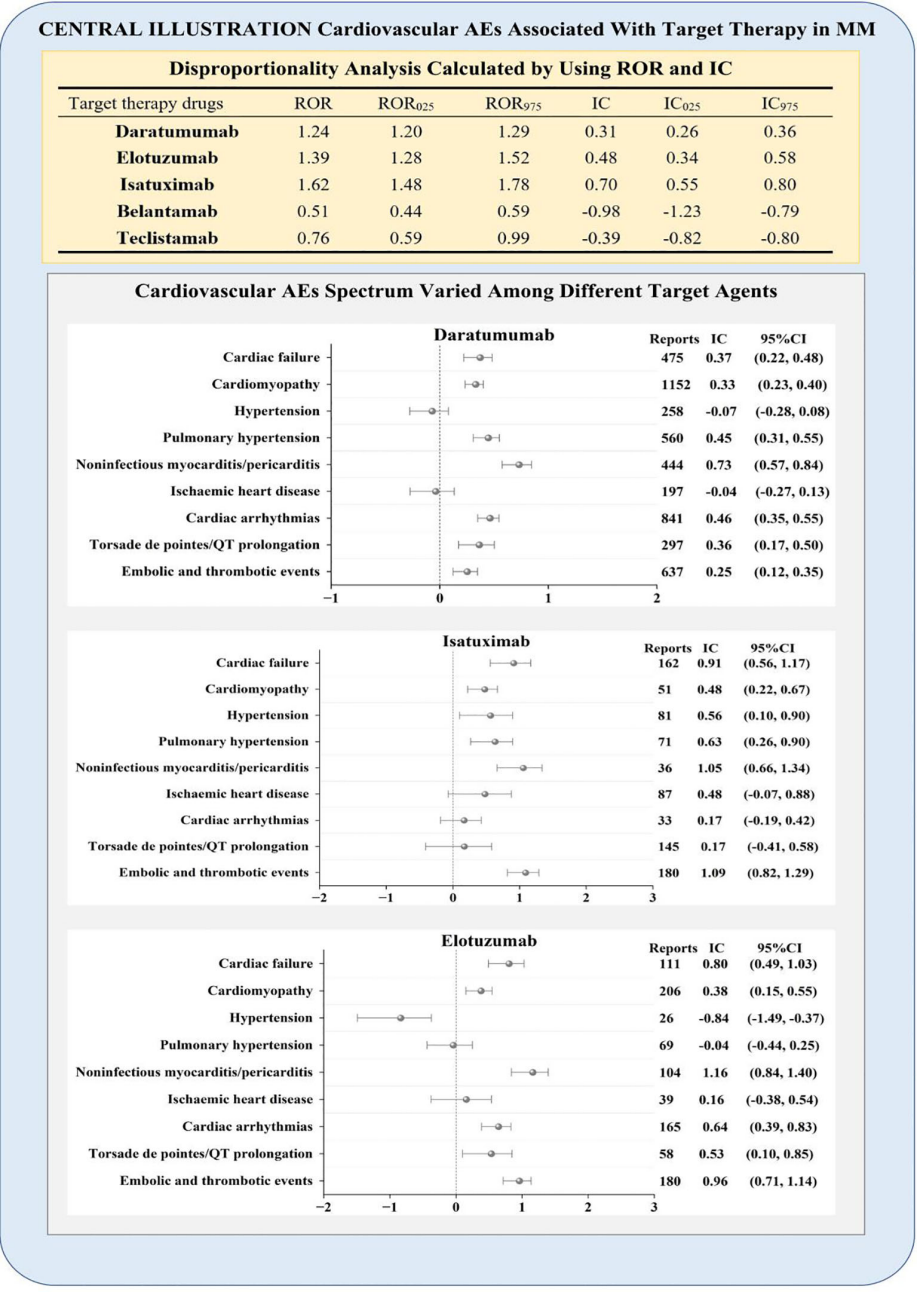
Central illustration. Disproportionality analysis calculated using ROR and IC. According to the disproportionality analysis, daratumumab, elotuzumab, and isatuximab exhibited significantly increased likelihood of being associated with reported cardiovascular AEs. AEs, adverse events; IC, information component; MM, multiple myeloma; ROR, reporting odds ratio.

A comparison of various targeted agents associated with cardiovascular AEs is presented in **Figure 2**.

Significant signal strength was observed in the cardiovascular AEs for daratumumab (IC_025_/ROR_025_ = 0.26/1.20), elotuzumab (IC_025_/ROR_025_ = 0.34/1.28), and isatuximab (IC_025_/ROR_025_ = 0.55/1.48). Nevertheless, for the antibody–drug conjugate belantamab (IC_025_/ROR_025_=−1.23/0.44) and bispecific antibody teclistamab (IC_025_/ROR_025_=−0.82/0.59), no significant associations were observed with cardiovascular AEs.

### Range of cardiovascular AEs based on SMQ for MM-targeted therapy drugs

As shown in **Table 3**, among the nine SMQ categories, cardiomyopathy (*N*=1,562, 23.1%) comprised the most frequently reported cardiovascular AE, followed by cardiac arrhythmias (*N*=1127, 16.7%), embolic and thrombotic events (*N*=989, 14.6%), pulmonary hypertension (*N*=744, 11.0%), cardiac failure (*N*=682, 10.1%), noninfectious myocarditis/pericarditis (*N*=632, 9.3%), and torsade de pointes/QT prolongation (*N*=395, 5.8%). Specifically, noninfectious myocarditis/pericarditis displayed the strongest signal value (IC_025_/ROR_025_ = 0.61/1.55), followed by cardiac arrhythmias (IC_025_/ROR_025_ = 0.29/1.23), embolic and thrombotic events (IC_025_/ROR_025_ = 0.28/1.23), cardiac failure (IC_025_/ROR_025_ = 0.27/1.22), pulmonary hypertension (IC_025_/ROR_025_ = 0.24/1.19), cardiomyopathy (IC_025_/ROR_025_ = 0.19/1.15), and torsade de pointes/QT prolongation (IC_025_/ROR_025_ = 0.11/1.10). However, ischemic heart disease and hypertension were not significantly associated with the MM-targeted therapy.

**Table 3:** Disproportionality analysis results for MM-targeted therapy associated with cardiovascular events based on specific SMQs.

Cardiovascular AE reports	Number (*n*)	ROR	ROR_025_	ROR_975_	IC	IC_025_	IC_975_
Cardiomyopathy	1,562	1.20	1.15	1.27	0.27	0.19	0.33
Cardiac arrhythmias	1,127	1.31	1.23	1.39	0.39	0.29	0.46
Embolic and thrombotic events	989	1.31	1.23	1.40	0.39	0.28	0.47
Pulmonary hypertension	744	1.28	1.19	1.38	0.36	0.24	0.45
Cardiac failure	682	1.32	1.22	1.42	0.40	0.27	0.48
Non-infectious myocarditis/pericarditis	632	1.67	1.55	1.81	0.74	0.61	0.83
Torsade de pointes/QT prolongation	395	1.21	1.10	1.34	0.27	0.11	0.40
Hypertension	346	0.90	0.814	1.006	−0.14	−0.32	−0.14
Ischemic heart disease	284	0.99	0.885	1.12	−0.00	−0.20	0.14

a IC, information component; MM, multiple myeloma; ROR, reporting odds ratio; SMQs, Standardized Medical Dictionary for Regulatory Activities Queries.

Importantly, analysis based on a single agent revealed varied patterns of cardiovascular AEs among daratumumab, elotuzumab, and isatuximab, as illustrated in **Figure 2**; cardiac failure, cardiomyopathy, noninfectious myocarditis/pericarditis, and embolic and thrombotic events were detected as signals for all three agents. Isatuximab was the only agent significantly associated with hypertension (IC_025_ = 0.10). Pulmonary hypertension was not associated with elotuzumab treatment (IC_025_=–0.44). Cardiac arrhythmias and torsade de pointes/QT prolongation were not associated with isatuximab (IC_025_=–0.19, 0.41).

**Figure 3:**
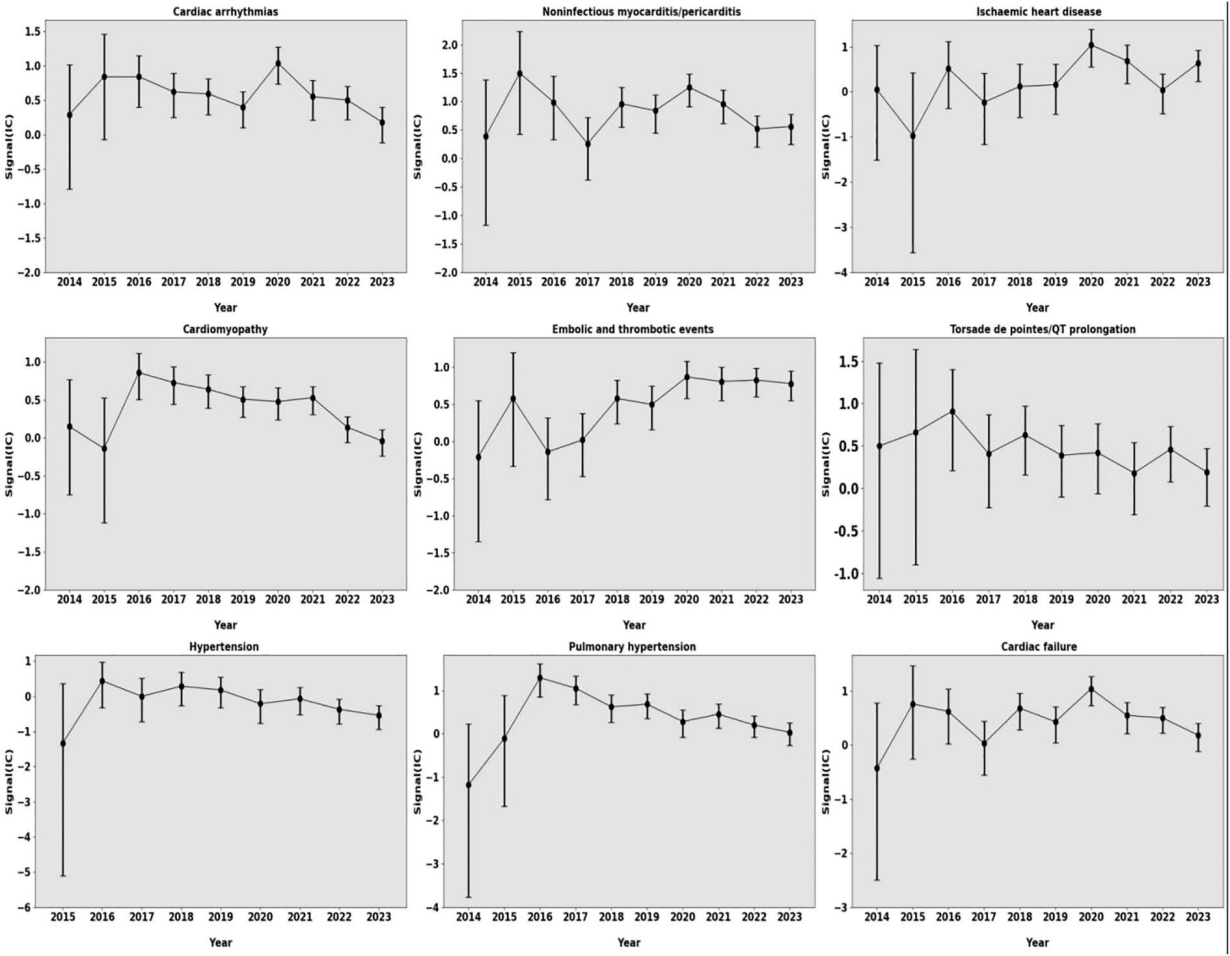
Information components over time for different types of cardiovascular AEs. AEs, adverse events.

Furthermore, we evaluated the pharmacovigilance signal intensities of these toxicities during the study period. Signal intensity fluctuated slightly from 2014 to 2023, and the CI gradually narrowed, indicating that pharmacovigilance signals maintained their stability, and these significant associations were consistently maintained (**Figure 3**).

### Range of cardiovascular AEs based on PT for MM-targeted therapy drugs

**Figure 4:**
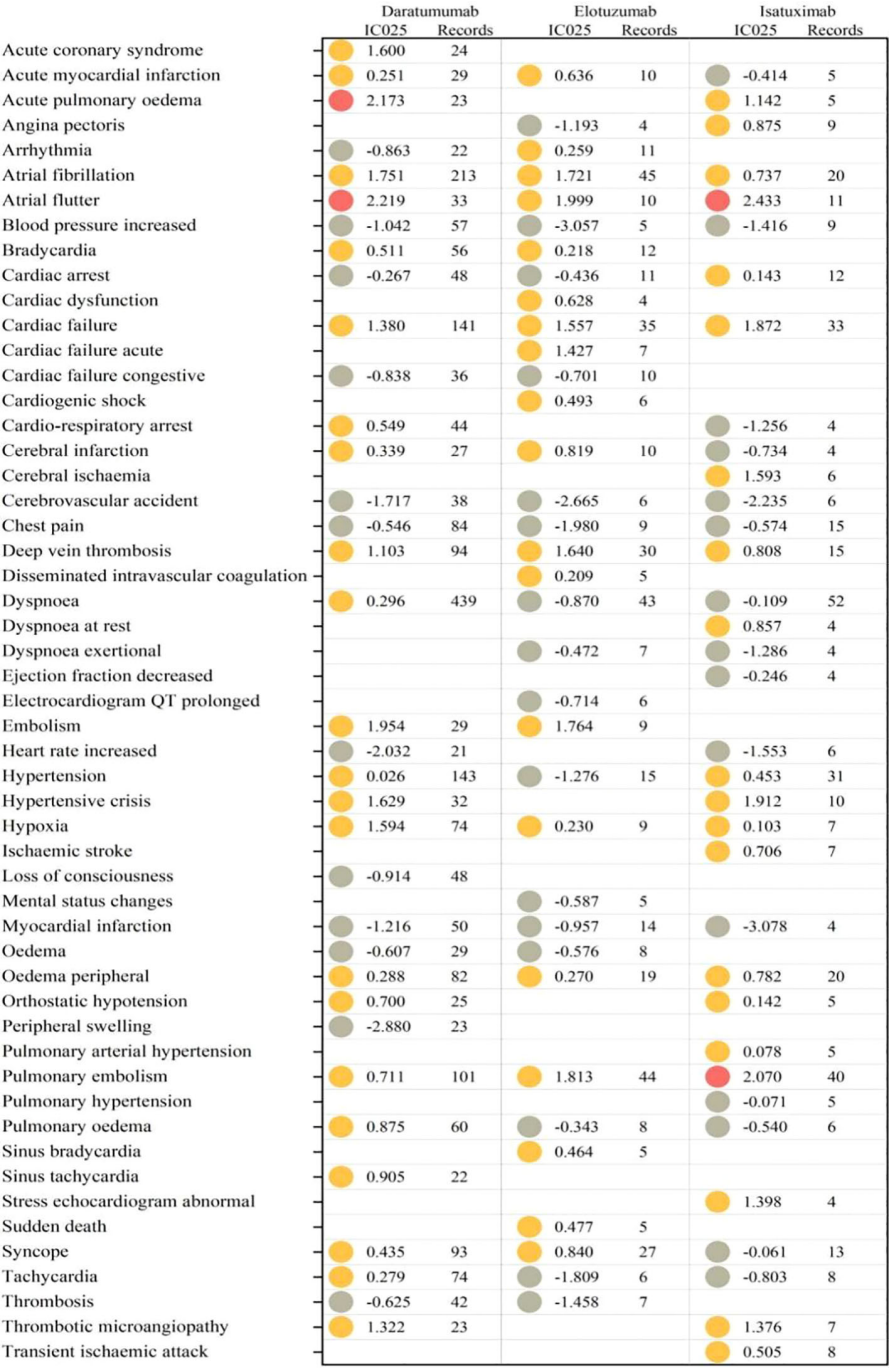
IC_025_ values for targeted therapy-related cardiovascular PTs. PTs, preferred terms.

We analyzed the top 35 (sorted by number of reports, with ≥3 reports) most frequently reported cardiovascular AEs in the PTs of daratumumab, elotuzumab, and isatuximab. A total of 23 PTs were significantly associated with daratumumab, 19 with elotuzumab, and 20 with isatuximab. Among these, seven PTs (atrial fibrillation, atrial flutter, cardiac failure, deep vein thrombosis, hypoxia, peripheral edema, and pulmonary embolism) exhibited significant correlations with all three agents, and atrial flutter had the strongest signal (IC_025_ = 2.433 for isatuximab, IC_025_ = 2.219 for daratumumab, and IC_025_ = 1.999 for elotuzumab (**Figure 4**).

For daratumumab, the five most common PTs were dyspnea (*N*=439, IC_025 =_ 0.296), atrial fibrillation (*N*=213, IC_025_ = 1.75), hypertension (*N*=143, IC_025_ = 0.03), cardiac failure (*N*=141, IC_025_ = 1.38), and pulmonary embolism (*N*=101, IC_025_ = 0.71).

For elotuzumab, the five most common PTs were atrial fibrillation (*N*=40, IC_025_ = 2.07), pulmonary embolism (*N*=33, IC_025_ = 1.87), cardiac failure (*N*=31, IC_025_ = 0.45), deep vein thrombosis (*N*=20, IC_025_ = 0.74), and syncope (*N*=20, IC_025_ = 0.78). Atrial flutter had the strongest signal (*N*=10, IC_025_ = 1.99).

For isatuximab, the five most common PTs were pulmonary embolism (*N*=27, IC_025_ = 0.84), cardiac failure (*N*=27, IC_025_ = 0.84), hypertension (*N*=27, IC_025_ = 0.84), atrial fibrillation (*N*=27, IC_025_ = 0.84), and peripheral edema (*N*=27, IC_025_ = 0.84).

### Onset and survival analysis for cardiovascular AEs of MM-targeted therapy drugs

**Figure 5:**
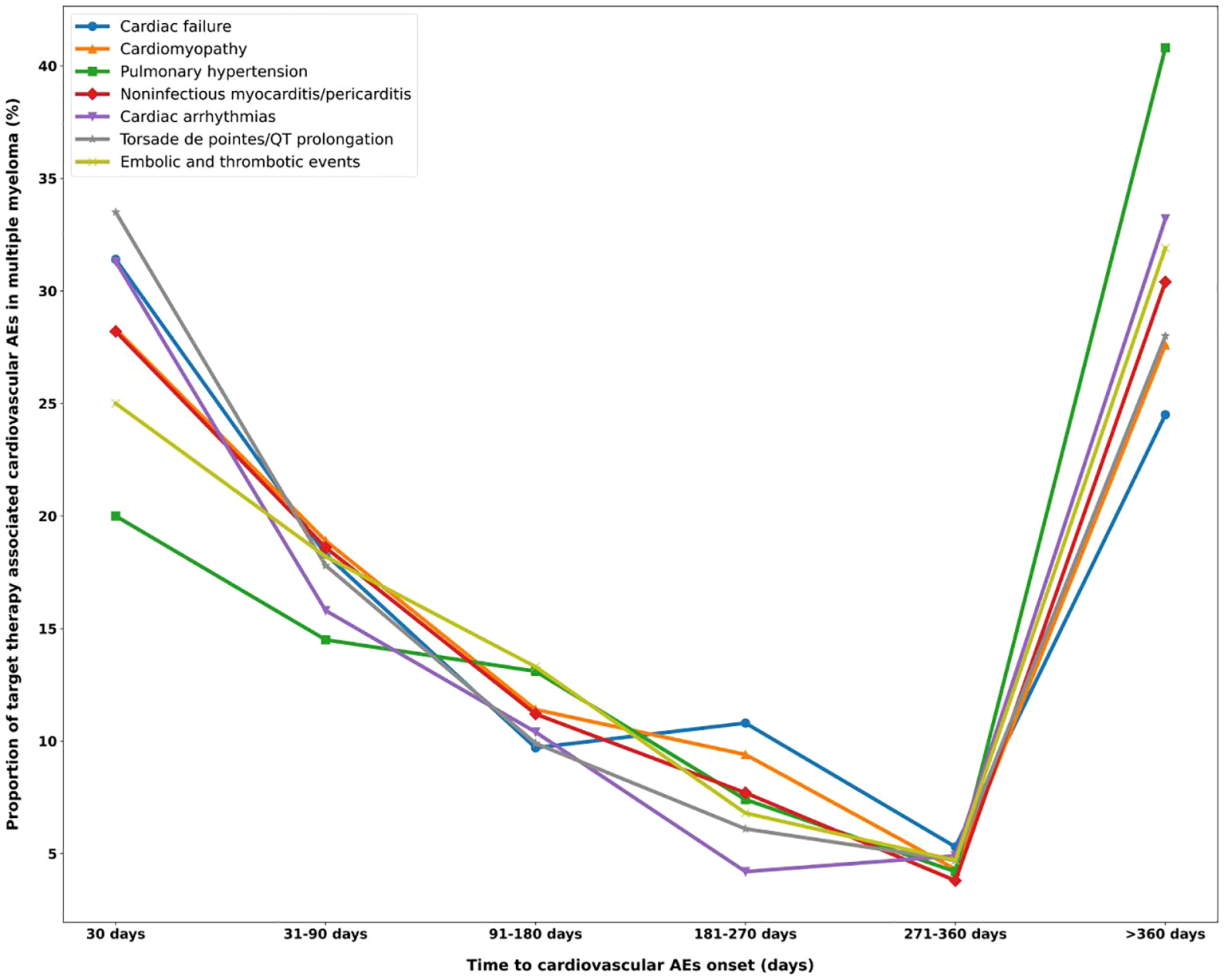
Time of onset for different categories of cardiovascular AEs. AEs, adverse events.

**Figure 6:**
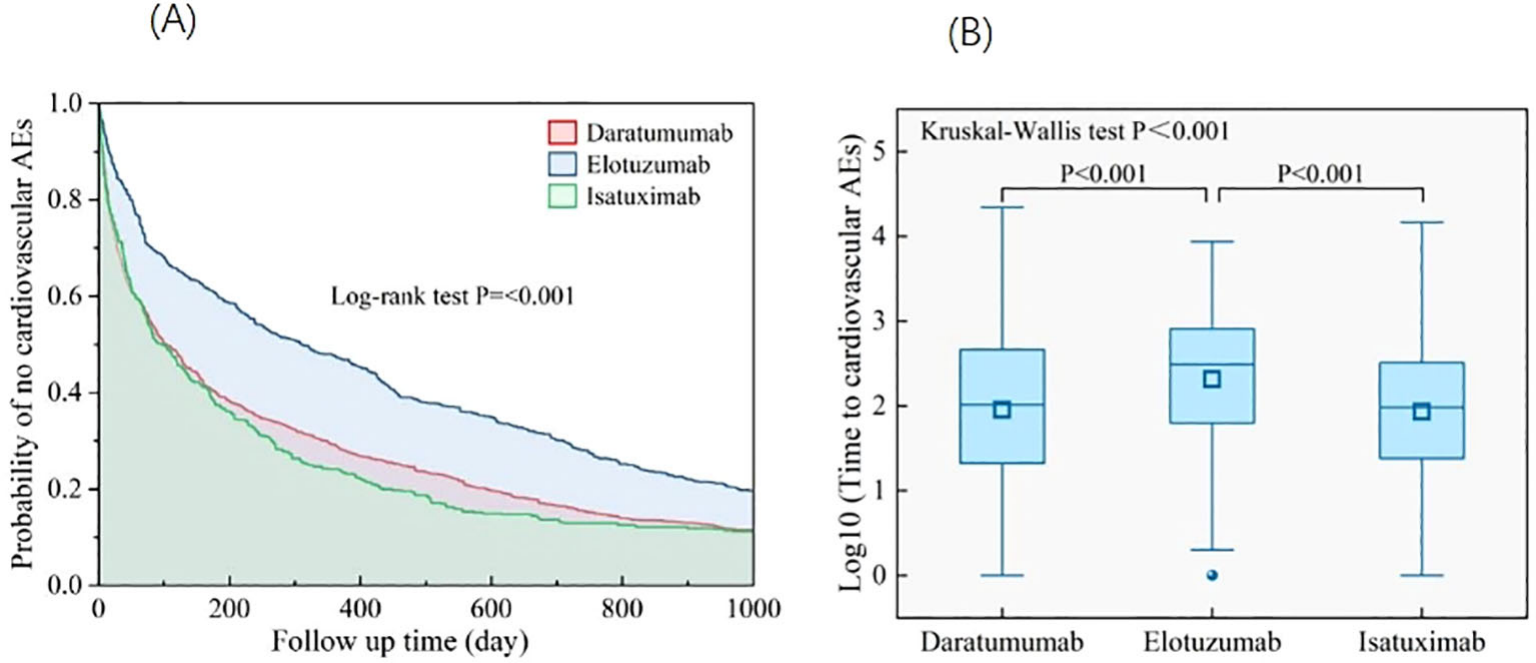
**(A)** Analysis of differences in onset of cardiovascular AEs among different targeted agents. **(B)** Kaplan–Meier curves for cardiovascular AEs in MM-targeted therapy. AEs, adverse events; MM, multiple myeloma.

More than half of the cardiovascular AEs occurred 6 months after initiation of targeted therapy, with a median onset of 129 days (interquartile range=27,528), except for pulmonary hypertension (**Figure 5**). Approximately 33.5% of torsade de pointes/QT prolongations occurred within 1 month of drug administration. The lowest ratio in the first month was 20% of pulmonary hypertension. A sustained decline was observed in the ratio of cardiovascular AE onset over time and reached the lowest at 271–360 days. Unexpectedly, at >360 days, a rebound in the ratio of cardiovascular AEs onset was observed, among which the ratio of pulmonary hypertension occurrence had the most prominent increase of approximately 40.8%. The onset for various targeted therapies (daratumumab, elotuzumab, and isatuximab) was compared. Kruskal–Wallis tests revealed that elotuzumab had a significantly later median AE onset of approximately 573 days (*p* < 0.001), which, for daratumumab and isatuximab, was approximately 407 and 427 days, respectively. The Kaplan–Meier curve operated with the log-rank test that showed that compared with daratumumab and isatuximab, elotuzumab exhibited a lower cardiovascular AE risk (*p* < 0.001) (**Figure 6**).

**Figure 7:**
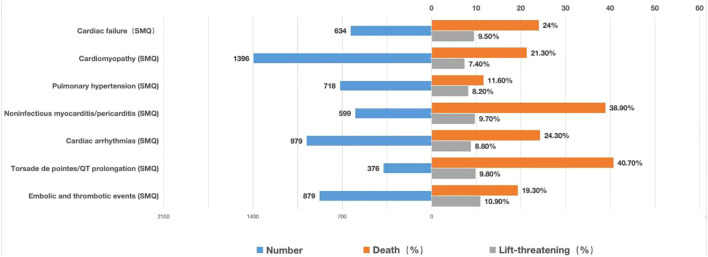
Records and proportions of death/life-threatening outcomes of cardiovascular AEs. AEs, adverse events.

Moreover, mortality due to various cardiovascular AEs was calculated and illustrated in a bar chart (**Figure 7**). Torsade de pointes/QT prolongation (40.7%) and noninfectious myocarditis/pericarditis (38.9%) contributed to a higher death outcome incidence, which is consistent with the findings that targeted therapies possess intense pharmacovigilance signals in torsade de pointes/QT prolongation and noninfectious myocarditis/pericarditis.

## Discussion

The ICARIA-MM (16), ELOQUENT (17, 18), and GRIFFIN (19) trials, which were conducted using a meticulous methodology, demonstrated that targeted therapy significantly mitigates MM progression and improves prognosis. Recognizing the efficacy of MM-targeted therapy, the FDA recently approved five targeted agents, namely, daratumumab, elotuzumab, isatuximab, belantamab mafodotin (20, 21), and teclistamab (22). However, studies on targeted therapy-induced cardiovascular AEs in patients with MM, using large-sample real-world data, are limited, and no studies provided data after stratifying by sex, age, cardiac profiles, and outcomes. This strongly underscores the need for continuous postmarketing surveillance.

To our knowledge, the present study is the most comprehensive pharmacovigilance study based on the FAERS database examining the association between MM-targeted therapy and cardiovascular AEs, including the range, onset, and outcomes of cardiovascular AEs, as well as offering an extensive comparison of such patterns between daratumumab, elotuzumab, and isatuximab. The main findings of our study are as follows:

### Cardiovascular AE burden and clinical characteristics of MM-targeted therapy drugs

Our research indicates that cardiovascular AEs accounted for 17.1% of all reported AEs associated with MM-targeted therapy. Moreover, annual cardiovascular AE reports are progressively increasing. Notably, daratumumab, elotuzumab, and isatuximab were significantly associated with an increased risk of cardiovascular AEs, as indicated by significantly higher signal values. These findings indicate that cardiovascular AEs may be noteworthy for MM-targeted agents. With continuous development and widespread application of targeted therapies, the incidence of targeted therapy-related cardiovascular AEs is expected to increase, underscoring the importance of paying earnest attention to this issue.

In the descriptive analysis, men and older patients accounted for a higher proportion of reported cardiovascular AEs. According to the Global Burden of Disease Study in 2016, after the age of 15 years, the incidence of MM increases steadily with age, and a higher incidence was observed in individuals aged >60 years. A 1.5-0 to 2-fold sex-specific difference in age-standardized incidence rates was identified, with men having a higher incidence than women across all age groups (23). These epidemiological data may explain this phenomenon; moreover, they suggest that more attention is required for screening and monitoring of cardiovascular AEs in male and older patients with MM.

### Cardiovascular AE profile of MM-targeted therapy drugs

This study revealed a broad range of cardiovascular AEs associated with MM-targeted therapy, with seven SMQs identified with significantly meaningful signals: cardiomyopathy, cardiac arrhythmias, embolic and thrombotic events, pulmonary hypertension, cardiac failure, noninfectious myocarditis/pericarditis, and torsade de pointes/QT prolongation. Cardiovascular AE profiles varied among different targeted agents.

Daratumumab was associated with the highest number of reported cardiovascular AEs in this study. Consequently, we evaluated the cardiovascular safety data from large daratumumab clinical trials. In the POLLUX trial, cardiovascular AEs in the daratumumab plus lenalidomide/dexamethasone group included peripheral edema (25.4%), dyspnea (23.7%), and syncope (5.7%), with a higher frequencies than in the lenalidomide/dexamethasone group (8). The CASSIOPEIA trial revealed a slightly higher incidence of hypertension in the daratumumab plus VTd group compared with the bortezomib, thalidomide, and dexamethasone (VTd) group (6% vs. 4%) (24). In the MAIA trial, the daratumumab group exhibited a higher incidence of pulmonary embolism and hypertension (25). In our study, the abovementioned cardiovascular AEs exhibited significant signals, except for hypertension. In addition, daratumumab was significantly associated with an increased noninfectious myocarditis/pericarditis risk (IC_025_ = 0.57), cardiac arrhythmias (IC_025_ = 0.35), pulmonary hypertension (IC_025_ = 0.31), cardiomyopathy (IC_025_ = 0.23), cardiac failure (IC_025_ = 0.22), torsade de pointes/QT prolongation (IC_025_ = 0.17), and embolic and thrombotic events (IC_025_ = 0.12). Noninfectious myocarditis/pericarditis exhibited the strongest signal value (IC_025_ = 0.57), although these reports were not the most common (*N*=444). Meanwhile, mortality was approximately 38.9% among reported noninfectious myocarditis/pericarditis cases, suggesting that greater attention is required regarding myocarditis/pericarditis screening and prevention. Although hypertension did not exhibit a significant signal, 258 daratumumab-induced hypertension reports were observed in this study, accounting for 5.3% of all daratumumab-related cardiovascular AEs. Therefore, during the clinical application of daratumumab, monitoring of blood pressure should not be ignored. Owing to limited sample size and population, cardiovascular AE profiles are not adequately reported in clinical studies. Postmarketing surveillance and real-world data can be used to determine associated cardiovascular AEs more objectively and comprehensively. The difference between real-world and clinical research data is notable.

Another CD38-targeted monoclonal antibody, isatuximab, was associated with a relatively small proportion of cardiovascular AEs, which may be related to its later FDA approval. Isatuximab possessed the strongest signal value for cardiovascular AEs, suggesting that more attention should be paid to monitoring cardiovascular AEs during isatuximab clinical administration. In the IKEMA trial, the incidence of hypertension was higher in the isatuximab group than in the control group (37% vs. 31%) (26). In the present study, isatuximab also exhibited a significant signal for hypertension, which is different from daratumumab and elotuzumab. In addition, embolic and thrombotic events had the strongest signal (IC_025_ = 0.82) among isatuximab-related cardiovascular AEs. This highlights the importance of thrombus and blood pressure monitoring during isatuximab administration.

Regarding elotuzumab, in the largest phase 3 clinical trial, 24% of patients presented with cardiac disorders; however, detailed data regarding cardiovascular events were not reported (18). This study described the spectrum of elotuzumab-related cardiovascular AEs. Noninfectious myocarditis/pericarditis exhibited the strongest signal (IC_025_ = 0.84), followed by embolic and thrombotic events (IC_025_ = 0.71), cardiac failure (IC_025_ = 0.49), cardiac arrhythmias (IC_025_ = 0.39), cardiomyopathy (IC_025_ = 0.15), and torsade de pointes/QT prolongation (IC_025_ = 0.10). Our findings address the gap regarding details of elotuzumab-related cardiovascular AEs.

Among the three targeted agents, atrial flutter exhibited the strongest signal among the cardiovascular PTs, which is consistent with a higher frequency of cardiac arrhythmias in the SMQ. MM arrhythmias are secondary to multifactorial arrhythmogenesis, including age, electrolyte disturbances, and cardiac amyloidosis, indicating that special attention needs to be paid to correcting reversible factors that can lead to arrhythmia in patients with MM treated with targeted drugs.

Collectively, these findings suggest differences in cardiovascular AEs with specific MM-targeted agents, warranting individualized cardiovascular monitoring programs for patients with MM (10). The mechanisms underlying cardiovascular AEs associated with isatuximab, elotuzumab, and daratumumab are poorly understood and require further investigation.

### Onset of cardiovascular AEs with MM-targeted therapy drugs

To our knowledge, no study has investigated cardiovascular AE onset with MM-targeted therapy, and this study is the first to compare the onset of specific cardiovascular AEs based on SMQs. Cardiovascular AE risk was higher within the first month and gradually decreased thereafter; however, it may increase rapidly again after 1 year. This trend was observed for all AEs. Especially for pulmonary hypertension, noninfectious myocarditis/pericarditis, cardiac arrhythmias, and embolic and thrombotic events, risk was higher after 1 year than during the first month. This finding suggests that MM-targeted therapy-related cardiovascular AE risk is a dynamic variable. Care and long-term follow-ups are necessary to cope with risk changes throughout the pathway.

Regarding the time from administration to occurrence of AEs, the onset of elotuzumab was significantly longer than that of daratumumab and isatuximab. In addition, the Kaplan–Meier curve showed that elotuzumab had a lower cardiovascular AE risk compared with that of daratumumab and isatuximab. This may be the reason for the number of cardiovascular AE reports for elotuzumab being significantly lower than that for daratumumab, although the reports were approved by the FDA simultaneously in 2015.

### Outcomes and survival related to cardiovascular AEs from MM-targeted therapy drugs

Regarding the outcomes of AEs, this study showed that most cardiovascular AEs were serious, and deaths or life-threatening events accounted for approximately 26.3%. Although torsade de pointes/QT prolongation had the smallest number of reports (*N*=376), torsade de pointes/QT prolongation had the highest mortality rate (40.8%) among the reported cardiovascular AEs. However, QT prolongation has not been reported in clinical studies on MM-targeted therapy (27); moreover, this condition is often ignored as it is asymptomatic in patients without ventricular arrhythmia. Therefore, electrocardiogram and QT evaluation must be emphasized before and during the application of targeted therapy in patients with MM.

### Limitations

Although FAERS data mining can serve as an effective auxiliary to clinical trials, some limitations of this study should be acknowledged. First, the FAERS database contains incomplete clinical data regarding history and clinical data. Second, the loss of reports or inaccurate reports are inevitable because the heterogeneous data sources of the FAERS have inconsistent processes for spontaneous reporting. Finally, all patients who received the drug were not included, which could lead to underreporting and therefore could not be used to calculate the incidence of cardiovascular AEs. Future studies should focus on converting the consequences of safety issue findings into practical clinical scenarios and address this deficiency.

## Conclusions

Targeted therapy in patients with MM is significantly associated with an increased risk of a diverse range of cardiovascular AEs. The range and outcomes of cardiovascular AEs vary among agents; moreover, data on these events differed from the findings of previous clinical studies. These insights offer valuable evidence for the precise management of cardiovascular AEs associated with MM-targeted therapy.

## Data Availability

The datasets presented in this study can be found in online repositories. The names of the repository/repositories and accession number(s) can be found below: The FDA Adverse Event Reporting System (FAERS) database.
